# Correlation of Cytokine Levels and Microglial Cell Infiltration during Retinal Degeneration in RCS Rats

**DOI:** 10.1371/journal.pone.0082061

**Published:** 2013-12-13

**Authors:** Yong Liu, Xuesen Yang, Tor Paaaske Utheim, Chenying Guo, Mingchun Xiao, Yan Liu, Zhengqin Yin, Jie Ma

**Affiliations:** 1 Southwest Hospital, Southwest Eye Hospital, Key Laboratory of Visual Damage and Repair of Chongqing, Third Military Medical University, Chongqing, China; 2 Institute of Tropical Medicine, Third Military Medical University, Chongqing, China; 3 Schepens Eye Research Institute, Massachusetts Eye & Ear Infirmary, Department of Ophthalmology, Harvard Medical School, Boston, Massachusetts, United States of America; 4 Department of Medical Biochemistry, Oslo University Hospital, Oslo, Norway; 5 College of Life Sciences, Southwest University, Chongqing, China; Massachusetts General Hospital and Harvard Medical School, United States of America

## Abstract

Microglial cells, which are immunocompetent cells, are involved in all diseases of the central nervous system. During their activation in various diseases, a variety of soluble factors are released. In the present study, the correlation between cytokine levels and microglial cell migration in the course of retinal degeneration of Royal College of Surgeons (RCS) rats was evaluated. MFG-E8 and CD11b were used to confirm the microglial cells. In the retina of RCS rats, the mRNA expression of seven genes (MFG-E8 and its integrins αυ and ß5, CD11b and the cytokines TNF-α, IL-1ß, and MCP-1) formed almost similar bimodal peak distributions, which were centred at P7 and P45 to P60. In contrast, in rdy rats, which comprised the control group, a unimodal peak distribution centred at P14 was observed. The gene expression accompanied the activation and migration of microglial cells from the inner to the outer layer of the retina during the process of degeneration. Principal component analysis and discriminant function analysis revealed that the expression of these seven genes, especially TNF-α and CD11b, positively correlated with retinal degeneration and microglial activity during retinal degeneration in RCS rats, but not in the control rats. Furthermore, linear regression analysis demonstrated a significant correlation between the expression of these genes and the activation of microglial cells in the dystrophic retina. Our findings suggest that the suppression of microglial cells and the blockade of their cytotoxic effects may constitute a novel therapeutic strategy for treating photoreceptor death in various retinal disorders.

## Introduction

Microglial cells are immunocompetent cells of the central nervous system. In all diseases of the central nervous system, microglial cells are involved which typically convert from their normal resting state to activated forms. During the activation, microglial cells undergo substantial morphological changes and release a variety of soluble factors, such as reactive oxygen species, growth factors, and cytokines. These factors influence the acute and chronic phase of disease, and are important during subsequent regeneration [1]. Microglial cells have also been shown to enter the retina in response to naturally occurring neuron death during embryonic and postnatal development [2], [3]. Soluble factors released from microglial cells have been found to be implicated in the degeneration of cultured photoreceptor cells [4]. Moreover, activated microglia-derived nerve growth factor can induce cell death in the developing retina [5].

In animals with retinal degeneration, migration of phagocytic cells into the outer nuclear layer (ONL) is usually observed in the early stages of photoreceptor injury [4]. Essner and Gorrin reported that the degeneration of the inner and outer retinal layers could produce substances, which initiate the migration of mononuclear cells into an inflammatory site in response to specific mediators [6]. They suggested that these migratory cells could either come from blood vessels or from cells in the retina. Remodelling of the actin cytoskeleton, a key mediator of cell chemotaxis, is essential for migration [7], [8]. In vivo studies indicate that microglial cells quickly rearrange their actin networks and form membrane ruffles and leading edges for process extension and chemotaxis for the response to infections in the brain [9], [10]. Another imaging study showed that microglial cells respond rapidly by extending their processes toward a laser ablation injury [11]. These studies indicate that the migration of immune cells is a critical step during the initial response to injury or inflammation in neural system.

In retinas of rats with inherited retinal dystrophy characterized by progressive degeneration of photoreceptors, immune cells infiltrate to the ONL and subsequently appear in the interphotoreceptor space, where they accumulate during the process of the disease [6]. The circadian shedding of the tips of photoreceptor outer segments (POS) and their engulfment by the adjacent retinal pigment epithelium (RPE) are vital to the survival of the retina. It is well known that the phagocytic defect in RCS rat is owed to a mutation in the Mertk gene, which leads to the lack of a cell surface receptor tyrosine kinase to clean off the shed POS [12], [13]. Interestingly, RPE of RCS rats dramatically increased POS binding in MFG-E8 (milk fat globule-EGF factor 8 protein) enriched microenvironment in vitro [14], which indicated that MFG-E8 may have a compensatory function to maintain engulfment of impaired RPE during retinal degeneration. A previous study demonstrated that MFG-E8 stimulates rhythmic POS phagocytosis by ligating apical αυβ5 receptors in the RPE [14]. MFG-E8 is a soluble glycoprotein known to regulate inflammation and immunity through mediating apoptotic cell clearance [15], [16]. Our previous study confirmed that MFG-E8 significantly contributed to apoptosis through microglial cells; augmentation or abrogation of MFG-E8 expression in mouse microglial cells dramatically enhanced or decreased the activities of apoptosis in vitro [16].

Cytokines are extracellular proteins that play important roles in regulating apoptosis of cells in response to injury or inflammation. In the affected tissues, the activated immune cells express chemoattractant receptors and adhesion molecules that control and direct migration in response to inflammatory cues by communicating through short-range cytokines and cell-cell contact [17], [18]. Cytokines also play important roles in initiating and directing microglial cell migration during retinal degeneration [19]. In animals with retinal degeneration, the early stages of photoreceptor injury are usually accompanied by migration of phagocytic cells into the ONL [4]. A significant increase in mRNA levels for TNF-α, IL-1ß and MCP-1 was found in retinal degeneration rats [20]. The role of chemoattractants causing the accumulation of microglial cells in the neurodegeneration remains unclear. LaVail and Battelle [21] identified two such factors, regional and pigmentary, that significantly influence the rate of photoreceptor degeneration [6]. Given these findings, it is important to quantify MFG-E8 and other key cytokine levels in order to understand the dynamic change and functions related to the behaviour of microglial cells in dystrophic retinas. In the present study, we hypothesize that the behaviour of microglial cells is tightly bound up with the expression of certain related genes, which play important roles in the migration and phagocytosis of microglial cells during retinal degeneration of RCS rats. New insights into the mechanisms that underlie photoreceptor apoptosis in retinal degeneration would be of clinical interest and could lead to new treatments [22].

## Materials and Methods

### Animals and Ethics

The RCS (retinal dystrophic) rats and RCS-rdy rats (abbreviated to rdy rats, non-retinal dystrophic, used as controls) used in this study were maintained in the animal facility of the Southwest Eye Hospital, the Third Military Medical University. The rooms had regulated light-dark cycles (12∶12 hr) adjusted with a light timer. Mice were supplied by the Animal Care Centre of Southwest Hospital. All rats used in the studies were sacrificed in a CO_2_ inhalation chamber. The study received written consent from the IACUC of the Southwest Hospital, the Third Military Medical University, Chongqing, China. All experiments were performed in accordance with the association for Research in Vision and Ophthalmology Statement for the Use of Animals in Ophthalmic and Vision Research. The participants provided their written consent and approved the consent procedure for our studies.

### Immunofluorescent Staining

To identify the origin of MFG-E8 positive cells, double staining (rabbit polyclonal anti-MFG-E8 and mouse monoclonal anti-CD11b, 1∶400 for both antibodies, Chemicon, USA) were applied to some sections. The eye balls (P14 and P30) were harvested and fixed in 4% paraformaldehyde solution for ≥1 hr. Cryostat sections (12 µm) were blocked in 1× PBS containing 10% of normal goat serum and 0.3% of Triton for 1 hr at room temperature and then incubated with primary antibodies overnight at 4°C. Cy3 and FITC (goat anti-rabbit and donkey anti-mouse, 1∶1000, Zhongshan Comp, China) were used as secondary antibodies. The sections were examined under a confocal microscope (LSM 510 Meta, Carl Zeiss Meditec, USA).

To evaluate the distribution of microglial cells in retina, cryostat retinal sections (12 µm) of RCS rats (P14, P45 and P90) were stained with rabbit polyclonal anti-MFG-E8 (1∶400) and Cy3 (goat anti-rabbit, 1∶1000). The staining procedure was the same as above. Images of the sections were taken under a confocal microscope (LSM 510 Meta). MFG-E8 positive cells were counted in a 2,000 µm^2^ area on each image. Fiji (Coloc 2) software was used to calculate the degree of co-localization between markers.

### Quantitative RT-PCR

The retinas of RCS and rdy rats were collected at P0, P3, P7, P14, P30, P45, P60 and P90 to evaluate the mRNA levels using quantitative RT-PCR (qRT-PCR). Total mRNA was extracted (RNeasy Mini kit, Qiagen, ML, USA) for cDNA synthesis (SuperScript III First-Strand Synthesis SuperMix, Invitrogen, CA, USA). The procedures of mRNA extraction and cDNA synthesis followed those provided by the manufacturers and the primers are listed in Table 1. Reactions were performed in a 25 µl eppendorf tube with 2 µl of cDNA, 0.3 µl of forward and reverse primer (10 pmol/µl), 10 µl of 2× Mix (full velocity SYBR green qPCR master mix, Stratagene) Taq and 7.4 µl of ddH_2_O. The procedure for real-time qRT-PCR included 5 min at 99°C, followed by 40 cycles of 15 s at 94°C, 30 s at 59°C, and 45 s at 72°C (Roche LC480, Roche Applied Science). All the qRT-PCR reactions were run in triplicate to yield averaged Ct values. Expression (evaluated as fold change for each target gene) was normalized to ß-actin in microglial cells following the well-established delta-delta method [23], [24]. A non-template control was included in the experiment to estimate DNA contamination of isolated RNA and reagents.

**Table 1:** Summary of primers.

Genes	Primers	Sequences
β-action	Forward	gttgacatccgtaaagacc
	Reverse	gactcatcgtactcctgct
MFG-E8	Forward	gggctactcgggcatccact
	Reverse	atatacacagacgaggcagaaatc
αυ	Forward	ggcgagcactgtttctccataa
	Reverse	cagattcatcccgaagataggc
β5	Forward	caacccacggtccatcatctc
	Reverse	atctcctgtggcgtcatctgg
CD11b	Forward	tctccatcaagattccagccagta
	Reverse	ctagcaccgctttctccaccac
TNF-α	Forward	cccctttatcgtctactcctca
	Reverse	gccactacttcagcgtctcg
IL-1β	Forward	ctgtgactcgtgggatgatgac
	Reverse	ggagaataccacttgttggctta
MCP-1	Forward	ccccactcacctgctgctact
	Reverse	ggcatcacagtccgagtcaca

### Statistical Analysis

All statistical tests were performed with Systat 12 and SigmaPlot 11. The Wilcoxon signed-rank test was used to compare gene expression and the population of microglial cell in different retinal layers. Normalized data of the seven gene expressions were subjected to principal component analysis (PCA) and discriminant function analysis (DFA). DFA was used to evaluate the expression pattern of individual genes. DFA is a statistical analysis which predicts a categorical dependent variable by means of one or more continuous variables. The model is built in a stepwise manner. Specifically, in each step, all variables among groups are evaluated to determine which will maximally discriminate to denote a root, which is an equation consisting of a linear combination of gene expression changes [25]–[27]. PCA was used to view the internal relationship between the expressions of different genes. The data were presented as mean ± SD. P<0.05 was considered as statistically significant. For co-localization experiments, Pearson’s and Manders’s coefficients were calculated with Fiji (Coloc 2) software (National Institute of Health).

## Results

### Microglial Features of MFG-E8 Positive Cells

**Figure 1:**
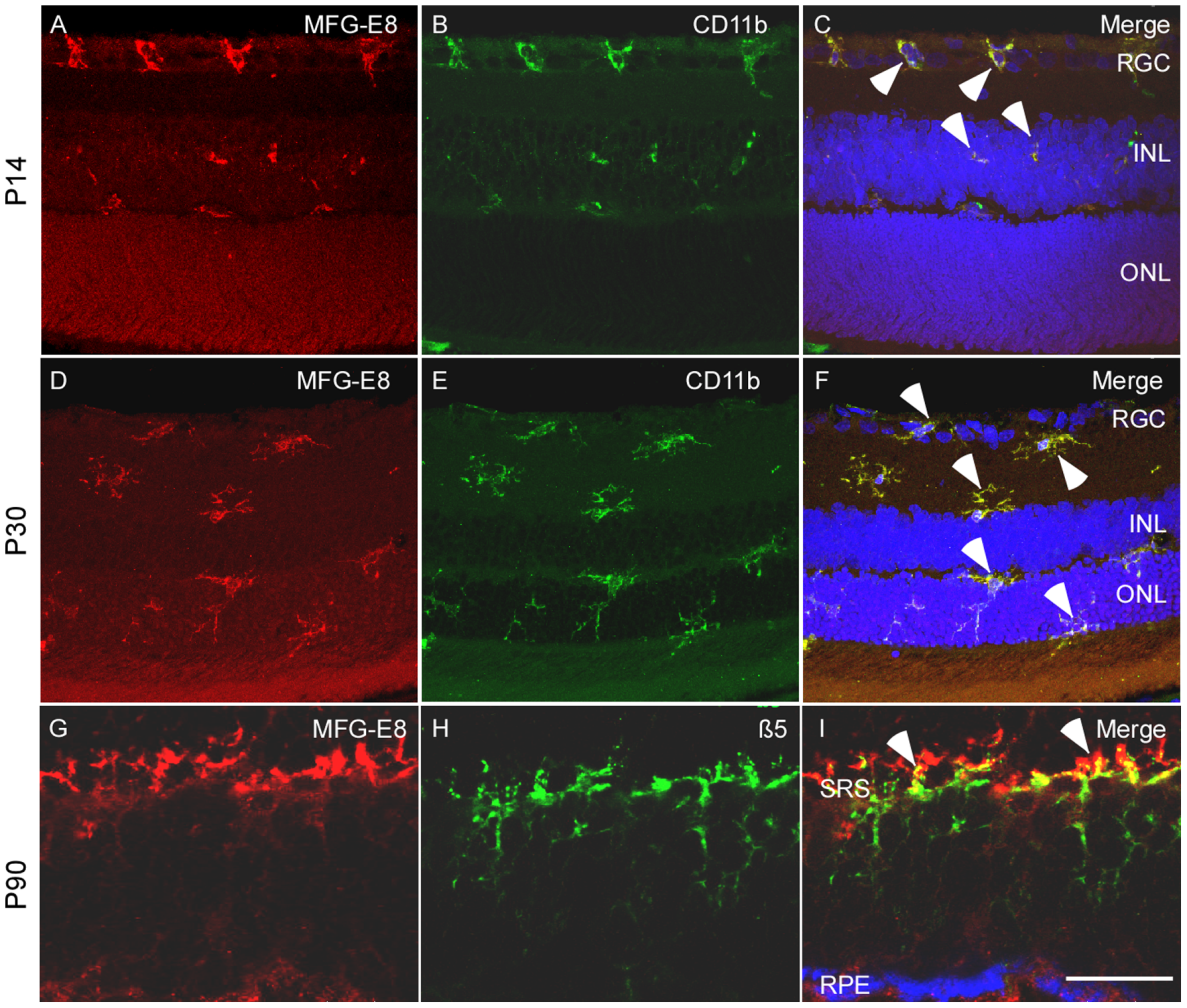
Microglial cell feature with two types of shape was identified in the MFG-E8 positive cells in the retina of the RCS rats. Double labelling was applied to detect the cell expressions MFG-E8 and CD11b. At P14 (A–C), most MFG-E8 labelled cells were found in the ganglion cell layer (GCL), with amoeboid shape (indicated by arrow heads). At P30 (D–F), MFG-E8 and CD11b positive cells show a well-ramified shape distributed in the whole retina (indicated by arrow heads). Double immunofluorescent staining indicates the expression of MFG-E8 (G) and ß5 integrin (H) in the retina of RCS rats at P90. (I) ß5 is mainly distributed in the subretinal space (SRS) and closely binds with MFG-E8 (indicated by arrow heads). Scale bar: 20 µm.

Some MFG-E8 positive cells in the outer plexiform layer (OPL) were observed engulfing nuclear fragments, which indicated that these cells could be involved in phagocytosis as microglial cells. To determine the properties of these cells, retina slices of P14 and P30 RCS rats were incubated with anti-MFG-E8 and anti-CD11b antibodies. The degree of co-localization of the expression of MFG-E8 and CD11b was calculated with Fiji (Coloc 2) software. Pearson’s R value based on a non-threshold picture was 0.91, whereas Manders M1 (non-threshold) and Manders M2 (non-threshold) were 0.99 and 0.94, respectively. This staining examination confirmed that these MFG-E8 positive cells were microglial cells (Figure 1A–1F). At P14 most of the microglial cells that assumed an amoeboid shape localized in the ganglion cell layer (GCL) (Figure 1A–1C). At P30, however, the microglial cells had acquired dendrite-like branches and were frequently observed in ONL (Figure 1D–1F).

αυß5 is an indispensable ligand for MFG-E8 during the process of phagocytosis by microglial cells. We investigated the relationship between MFG-E8 and ß5 in advanced retinal degeneration in RCS rats. The degree of co-localization between anti-ß5 and anti-MFG-E8 antibodies was calculated with Fiji (Coloc 2) software. Pearson’s R value based on a non-threshold picture was 0.92, whereas Manders M1 (no threshold) and Manders M2 (no threshold) were 0.99 and 0.98, respectively. These objective data clearly demonstrate the relationship between ß5 integrin and MFG-E8 in OPL at an advanced stage of retinal degeneration (Figure 1G–1H).

### Distribution and Migration of Microglial Cells

**Figure 2:**
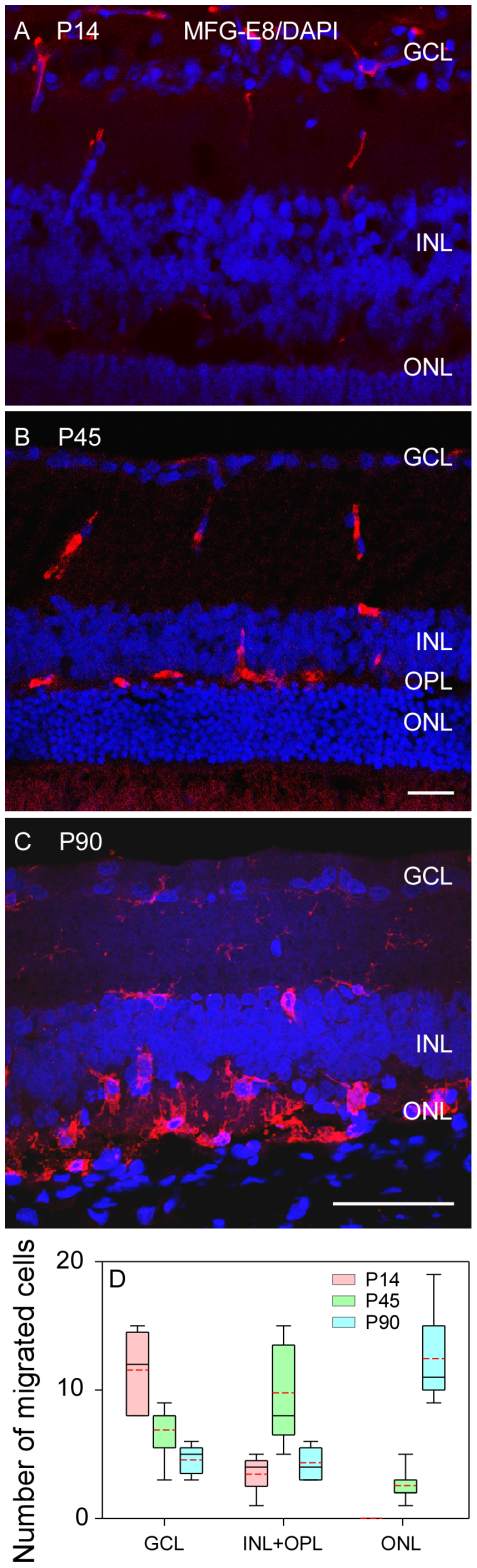
The distribution of MFG-E8 positive cells in the ganglion cell layer (GCL) to the outer layers of the retina during retinal degeneration in RCS rats. (A) MFG-E8 positive cells are mainly scattered in GCL at P14. (B) At P45, MFG-E8 positive cells were detected in the entire inner retina and outer plexiform layer (OPL). (C) At P90 most of the labelled cells were observed among the remaining photoreceptor cells. (D) The quantified distribution of MFG-E8 positive cells from the inner retina layer to the outer space. Scale bar: 50 µm.

A clear change in the distribution of MFG-E8 positive cells, which were confirmed as microglial cells (Figure 1), was observed in the retinal sections at P14, P45 and P90 of RCS rats. Specifically, in P14 rats most of the MFG-E8 positive cells were found in GCL (75%,12/16) and a few (25%, 4/16) in the inner nuclear layer (INL) (Figure 2A) (P<0.001). At P45, more MFG-E8 positive cells were found in INL (50%, 10/20) compared with GCL (35%, 7/20) (P = 0.055) and the outer nuclear layer (ONL, 15%, 3/20) (P<0.001) (Figure 2B). At P90, photoreceptor degeneration was more advanced, showing distinctive boundaries among layers of the blurred retina. At this stage, MFG-E8 positive cells were mostly found in the ONL (58%,13/23) but could also be observed in GCL (21%, 5/23) and INL (21%, 5/23) (both P<0.001) (Figure 2C). These findings indicate that MFG-E8 positive cells migrated from the inner retina layers to the ONL during retinal degeneration. Quantification of migrated cells is presented in Figure 2D.

### mRNA Expression of Microglial Related Factors

**Figure 3:**
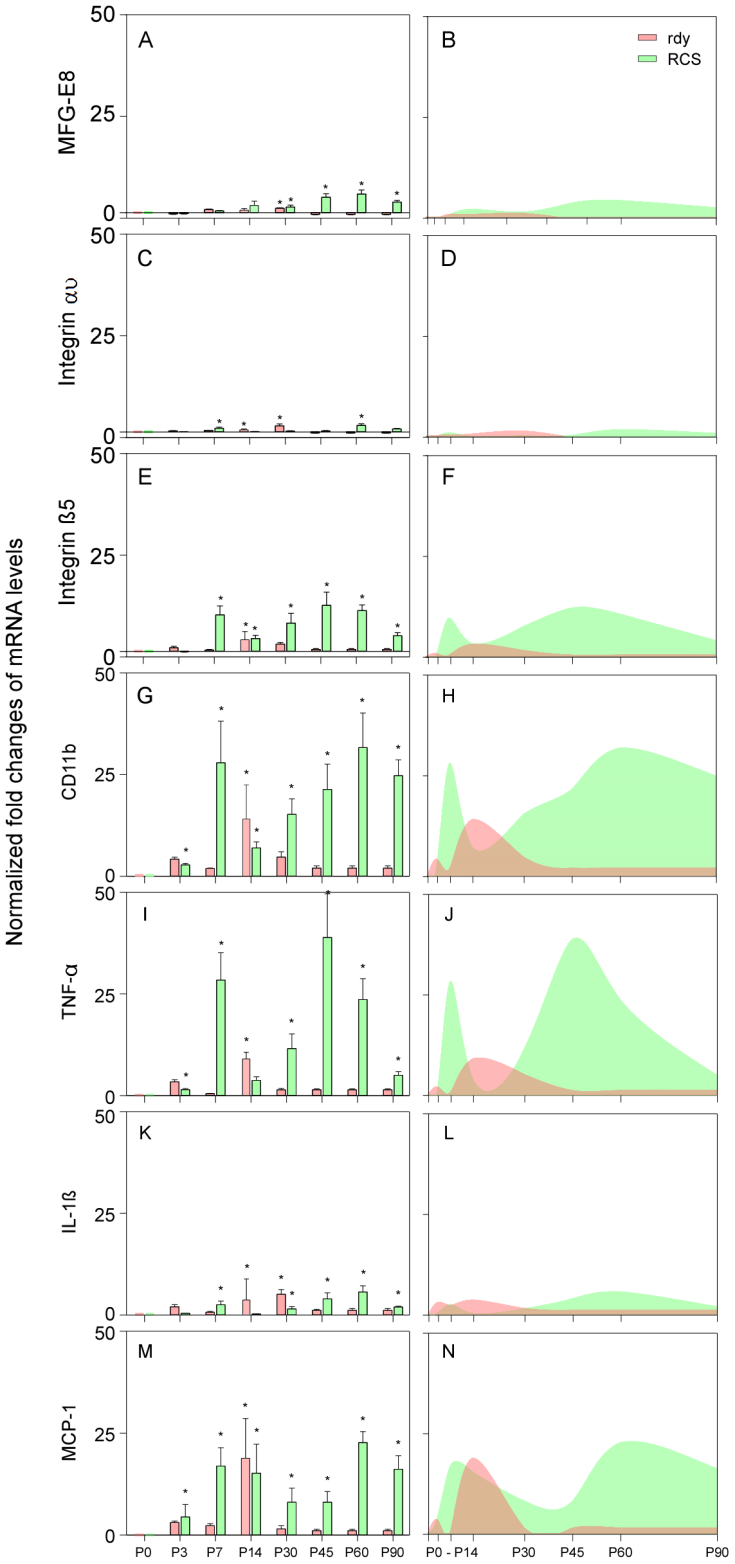
The mRNA expression of MFG-E8 and its integrins (αυ and ß5) and inflammatory factors in retinas of RCS and rdy rats. (A–N) all of the gene expression of RCS rats formed a bimodal peak pattern centred at P14 and P45 to P60 but formed a unimodal pattern centred at P14 in rdy rats. The mRNA levels of MFG-E8, integrin αυ and IL-1β were relatively lower than the others in both rats, although the expression of integrin β5, CD11b, TNF-α and MCP-1 were quite high in the two rats, especially in RCS rats (E–J and M–N). *P<0.05.

We initially characterized the mRNA expression pattern of MFG-E8 and its integrins (αυ and ß5), which are closely related to the phagocytosis process of microglial cells on apoptotic cells, in the retinas of rdy and RCS rats from P0 to P90. In the control rdy rat, MFG-E8, integrin αυ, and integrin ß5 mRNA expression were low from P0 to P7. A modest expression peak was found at P14, which was significantly higher than the expression at P0 for each of the genes (Figure 3A, 3C and 3E, all P<0.05). The expression began to decrease from P30 and returned to baseline from P45 (Figure 3A, 3C and 3E). Thus, mRNA expression of the three genes showed a unimodal peak distribution centred at P14 during the period in rdy rats (Figure 3B, 3D and 3F). Interestingly, in RCS rats, the expression of these three genes showed a modest bimodal peak distribution presenting at P7 and P45 to P60 (Figure 3B, 3D, and 3F). Both of the peak values were significantly higher than that of P0 for the three genes (Figure 3A, 3C and 3E, all P<0.05). The expression remarkably decreased from P60 for each of the three genes, but was still higher than P0 at P90 (Figure 3B, 3D, and 3F, all P<0.05). The expression of the three genes in RCS rats was in general significantly higher than those of rdy rats (Figure 3A–3F, all P<0.05).

To further evaluate the change of microglial characteristics in rdy and RCS rats during the retinal degeneration, mRNA of CD11b, TNF-α, IL-1ß and MCP-1 was measured from P0 to P90. In rdy rats, the expression of mRNA markedly increased at P14 compared with P0 and formed a unimodal peak distribution pattern, then returned to baseline level from P30 (Figure 3G–3N). The expression pattern of the four genes was similar to that of MFG-E8, integrin αυ and integrin ß5 in rdy rats. In RCS rats, the expression of the four genes showed a similar trend to that of the other three genes expressed in the previous section, forming a bimodal peak distribution centred at P7 and P45 to P60, respectively (Figure 3G–3N). Generally, the expression from P7 was significantly higher than that of P0 for the four genes (Figure 3); and the expression of the four genes in RCS rats was for the most part significantly higher than those of rdy rats, with a notable exception at P14 (Figure 3).

### Expression Trend of Genes Related to Microglial Cells

**Figure 4:**
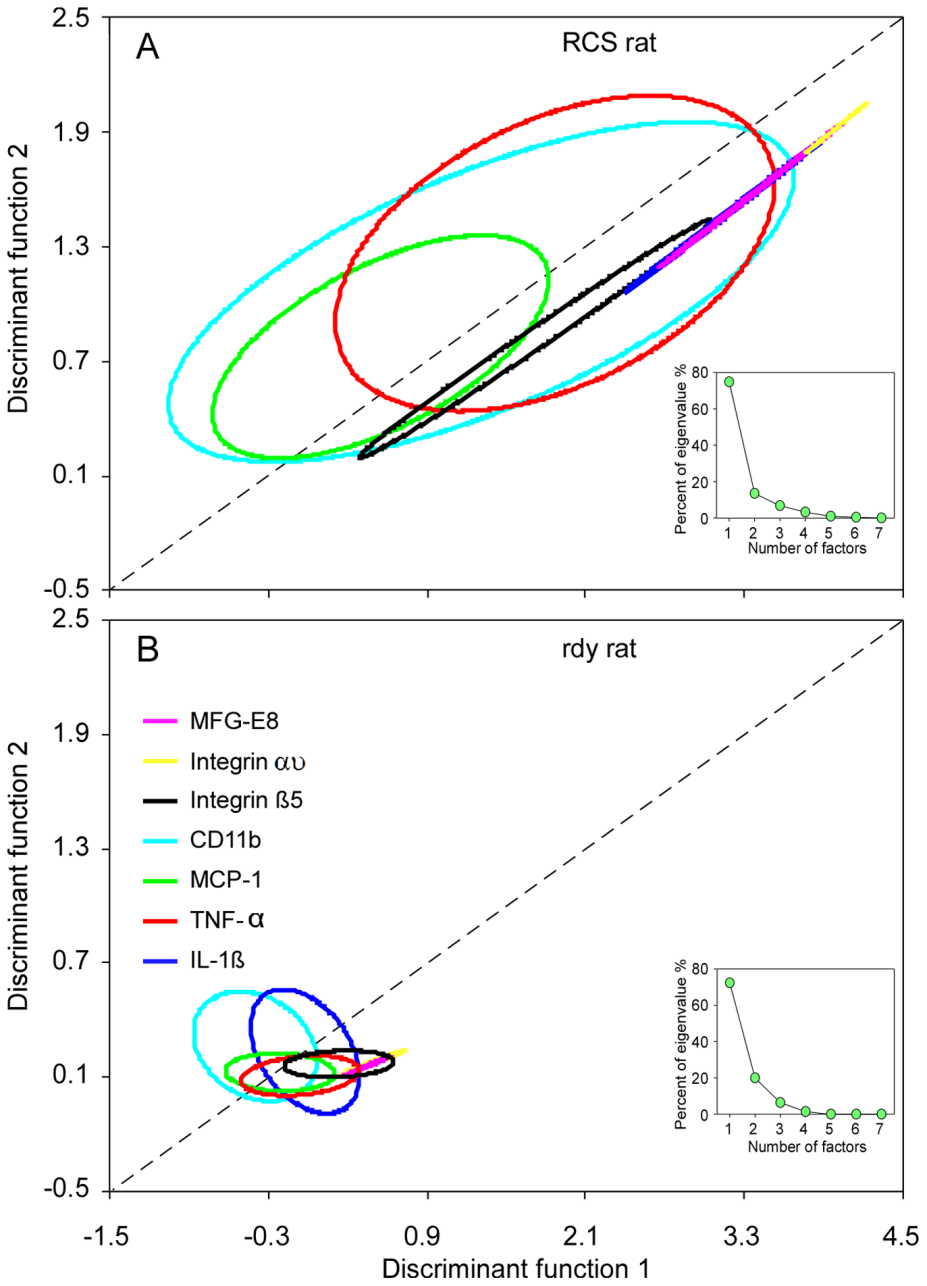
The mRNA expression patterns in the retinas of RCS and rdy rats. (A–B) The 75% centroid of confidence ellipses based on the discrimination function analysis indicated the similarity of mRNA expression patterns in RCS and rdy rats, respectively. The mRNA expression trend showed a positive association with retina development from P0 to P90 in RCS rats (A) but it was relatively stable in rdy rats (B).

qRT-PCR measurements of the seven genes from P0 to P90 were weighted with PCA. Eighty per cent of the eigenvalue was contributed by two (TNF-α, 75%; CD11b, 14%) of the seven genes (inserts in Figure 4A–4B). Therefore, a two-dimensional plot was generated from the matrix distance of gene expression measured by DFA. In RCS rats, the 75% centroid of confidence ellipses based on DFA has a similar direction to their major axis with positive slope. Specifically, the expression showed positive association between discriminant functions 1 and 2 (Figure 4A). This finding showed that the expression of the seven genes was (to some extent) associated with retinal degeneration, which confirmed the increased activity of microglial cells in the course of retinal degeneration in RCS rats. In the control rdy rats, however, the 75% centroid of confidence ellipses based on DFA showing the expression of the seven genes was quite stable or slightly depressed (Figure 4B).

### Correlation of mRNA Levels and Migratory Microglial Cells

**Figure 5:**
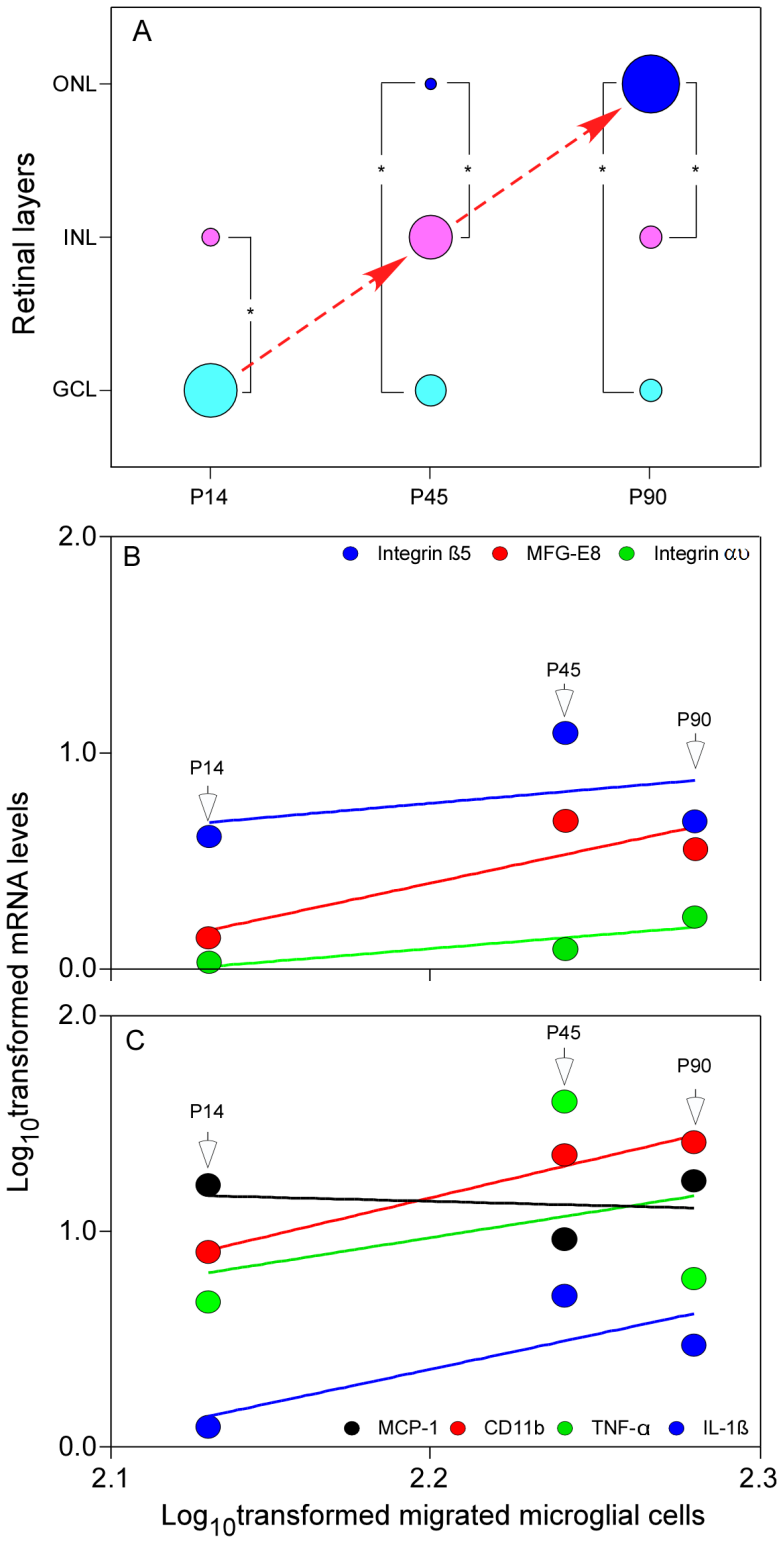
The distribution of migratory microglial cells and the association between mRNA levels and the number of migrated microglial cells during retinal degeneration in RCS rats. (A) The distribution of microglial cells in the retina of RCS rats from GCL to ONL during retinal degeneration from P14 to P90. (B) mRNA levels of MFG-E8 and integrin αυ showed more significant linear regression with the migration of microglial cells than the integrin β5. (C) mRNA level of CD11b was significantly associated with the migration of microglial cells and IL-1β and TNF-α showed a modestly linear regression, but the association between mRNA levels of MCP-1 and microglial cells was slightly negative, which might be owed to a dramatic reduction of apoptotic cells and a possible decrease in the phagocytic activity (or requirement) of microglial cells in the ONL.

The distribution of microglial cells (MFG-E8 positive cells) showed a dynamic change from GCL to ONL during the retinal degeneration of RCS rats. At P14, the population of microglial cells in GCL was bigger than that in INL (P<0.05, Figure 5A). More microglial cells were found in INL than CGL and ONL at P45 (Figure 5A). At P90, the retinal morphology of RCS rats showed significant alterations compared with the early stage; specifically, the ONL became quite thin (Figure 2C). Meanwhile, the population of microglial cells in ONL was remarkably larger than that in GCL and INL (Figure 5A, both P<0.001). These observations indicated that the microglial cells migrated from GCL to ONL during the retinal degeneration in RCS rats.

To investigate the relationship between gene expression and microglial cell distribution, log_10_-transformation was applied to them at P14, P45 and P90 in RCS rats. Linear regression analysis confirmed that microglial cell distribution was positively related to the expression of MFG-E8 and its integrin αυ, CD11b and inflammatory factor IL-1β (all R^2^>0.6). The expression of β5 and TNF-α showed a modest association with the distribution of microglial cells (R^2^<0.2), whereas MCP-1 showed a slightly negative correlation (Figure 5B–5C). Collectively, these observations suggest that the distribution of microglial cells is correlated with the expression of certain factors.

## Discussion

Microglial cells play an important role in keeping tissue healthy through phagocytosis of apoptotic cells. The ‘eat me’ signals on the surface of apoptotic cells attract microglial cells and initiate the process of phagocytosis [16]. In this study, we focused on investigating the relationship between the migratory behaviour of retinal microglial cells and the mRNA levels of MFG-E8 including its integrins (αυ and ß5) and a few key cytokines during retinal degeneration of RCS rats. We expected that there would be a concomitant change in the expression levels of some factors related to phagocytosis and inflammation. Therefore, we evaluated the mRNA levels of some typical genes and the dynamic change of retinal microglial cells in RCS rats.

PCA was used to estimate the potential relationship of the seven genes with microglial activity. PCA revealed that TNF-α majorly contributed to the root estimation (75%) followed by CD11b (14%) in RCS rats. This finding may indicate that the inflammatory effect (high expression of TNF-α) leads to the subsequent aggressive migration of microglial cells. We found that most microglial cells present an amoeboid shape in the early stage of retinal degeneration (P14), but assume a ramified shape at P30. This observation demonstrates that retinal microglial cells undergo significant morphological changes in activated condition. This can affect a variety of receptors and secreting soluble factors, which contribute to recognition and phagocytosis of dying or malfunctioning photoreceptors [28]. The major population of microglial cells clearly migrated from the GCL at P14, and moved to the INL at P45 before reaching ONL at P90. This observation indicates that microglial cells gradually accumulated in ONL with the development of photoreceptor apoptosis, which coincides with previous findings [6], [29]–[33]. The accumulation of microglial cells within the zone of photoreceptor degeneration probably correlates with the ability of these cells to recognize and destroy ‘sick’ neurons within the retina [34]. This process has been thought to involve some cytokines leading microglial cells to injury or infection areas [35]. As regards the occurrence of maximum microglial cells, the time point might differ in different studies. In a previous study involving RCS rats, proliferating retinal microglial cells found the maximum amount of microglial cells occurred at P21 [36]. Another study found that microglial cells were first seen in the retina at P7 and reached the maximum at P14 in the outer layers of the retina [34]. These studies do not agree with our observation that the robust microglial cells were observed after P45, which may be owed to the different sampling times.

Interestingly, we found that the mRNA levels of MFG-E8 including its integrins, CD11b (a typical microglial cell marker) and other key cytokines accompanied changes in the behaviour of microglial cells during retinal degeneration. mRNA levels of MFG-E8 and its integrins showed a similar bimodal peak pattern to that of microglial cells releasing factors (CD11b, TNF-α, IL-1β and MCP-1). These factors are strongly related to inflammatory response. Apoptosis showed increasing labelling of photoreceptor cells with progression of retinal dystrophy [37]. Therefore, the changes of their mRNA levels in the retina may reflect the activation of microglial cells during photoreceptor dysfunction in RCS rats. In addition, DFA analysis also revealed that the expression of the seven genes in RCS rats has a similar pattern. This confirms the increased activity of microglial cells during retinal degeneration. In the control rats, DFA tests showed that the expression of these seven genes was unchanged or slightly depressed.

Microglial cells may be involved in photoreceptor cell death in RCS rats [29]. This has been considered as an inadequate microglial cell response to control the injury that leads to the ensuing photoreceptor cell death. An in vitro examination found that microglial cells release soluble products that induce degeneration of cultured photoreceptor cells [4]. Therefore, activated microglial cells may secrete cytotoxic factors that promote photoreceptor cell death in dystrophic retinas. Previous studies proved that the production of nitric oxide and reactive oxygen species is a major source of some cytokines, including TNF-α, that can cause tissue damage after activation of phagocytic cells [36]. Thus, activated microglial cells are fully capable of releasing cytotoxic molecules that can induce continuous photoreceptor cell death in diseased retinas [4]. On the basis of the log-transformed gene expression and the number of migratory microglial cells, we found that the expression of six genes, excluding MCP-1, positively correlated with the number of migrated microglial cells from P14 to P90. This finding indicates that the increased number of migratory microglial cells in the outer layer of the retina was accompanied by increased gene expression produced by microglial cells.

There are some retinal diseases in which microglial cells play an important role. Consequently, microglia-targeted pharmacotherapy may be beneficial for neuron survival and regeneration [38]. For example, inhibition of microglial cell invasion in the photoreceptor cell layer may promote the survival of photoreceptor cells in the dystrophic retina. Therefore, the accumulation of outer segment debris may provide a signalling mechanism for the activation and chemotaxis of microglial cells. A genetic defect in the phagocytosis of rod outer segments by RPE cells may promote initial injury to the photoreceptors, but microglial cells may eventually be responsible for the induction of photoreceptor cell death [29]. Thus, suppression of microglial cells and blockade of cytotoxic effects may represent alternative new therapeutic strategies for treating photoreceptor death in the context of various retinal diseases.
